# Supplementary material for: Clinicopathological characteristics and prognosis analysis of gastrinoma based on the SEER database
Source: PLoS One. 2025 Dec 17;20(12):e0337940. doi: 10.1371/journal.pone.0337940 (PMC12711040; doi:10.1371/journal.pone.0337940)
Supplement: S1 Table — (DOCX) [file pone.0337940.s001.docx]

**Supplementary Table 1.** Baseline characteristics of patients with gastrinoma based on the SEER database before multiple imputation

| **Variables** | **N=160** | **%** |
| --- | --- | --- |
| **Marital status** |  |  |
| Married | 80 | 50.0 |
| Unmarried | 71 | 44.4 |
| Unknown | 9 | 5.6 |
| **Age, yesrs** |  |  |
| <29 | 5 | 3.1 |
| 30-39 | 14 | 8.8 |
| 40-49 | 25 | 15.6 |
| 50-59 | 40 | 25.0 |
| 60-69 | 48 | 30.0 |
| >69 | 28 | 17.5 |
| **Race** |  |  |
| White | 124 | 77.5 |
| Black | 27 | 16.9 |
| Others | 9 | 5.6 |
| **Location** |  |  |
| Stomach | 17 | 10.6 |
| Duodenum | 52 | 32.5 |
| Pancreas | 84 | 52.5 |
| Others | 7 | 4.4 |
| **Grade** |  |  |
| Well | 48 | 30.0 |
| Moderately | 11 | 6.9 |
| Poorly | 2 | 1.3 |
| Unknown | 99 | 61.9 |
| **Sex** |  |  |
| Male | 77 | 48.1 |
| Female | 83 | 51.9 |
| **T stage** |  |  |
| T1 | 19 | 11.9 |
| T2 | 22 | 13.8 |
| T3 | 7 | 4.4 |
| T4 | 4 | 2.5 |
| TX | 21 | 13.1 |
| Unknown | 87 | 54.4 |
| **N stage** |  |  |
| N0 | 32 | 20.0 |
| N1 | 35 | 21.9 |
| NX | 6 | 3.8 |
| Unknown | 87 | 54.4 |
| **M stage** |  |  |
| M0 | 51 | 31.9 |
| M1 | 22 | 13.8 |
| Unknown | 87 | 54.4 |
| **Surgery** |  |  |
| Yes | 97 | 60.6 |
| No | 61 | 38.1 |
| Unknown | 2 | 1.3 |
| **Chemotherapy** |  |  |
| Yes | 13 | 8.1 |
| No/Unknown | 147 | 91.9 |
| **Tumor size, cm** |  |  |
| ≤2.0 | 49 | 30.6 |
| 2.1-4.0 | 17 | 10.6 |
| >4.0 | 22 | 13.7 |
| Unknown | 72 | 45.0 |
| **Liver metastasis** |  |  |
| No | 54 | 33.8 |
| Yes | 18 | 11.3 |
| Unknown | 88 | 55.0 |
| **Lung metastasis** |  |  |
| No | 70 | 43.8 |
| Yes | 2 | 1.3 |
| Unknown | 88 | 55.0 |

Seer: surveillance, epidemiology, and end results
