# Supplementary material for: Clinicopathological characteristics and prognosis analysis of gastrinoma based on the SEER database
Source: PLoS One. 2025 Dec 17;20(12):e0337940. doi: 10.1371/journal.pone.0337940 (PMC12711040; doi:10.1371/journal.pone.0337940)
Supplement: Attachment — Submitted filename: Point by Point Response R1.docx [file pone.0337940.s003.docx]

Dear editor and reviewers

Submission ID PONE-D-25-00053

Title: Clinicopathological Characteristics and Prognosis Analysis of Gastrinoma Based on the SEER Database

By: zhenguo qiao et al

Thank you very much for your letter and for the editors’ and reviewers’ comments concerning our manuscript entitled “Clinicopathological Characteristics and Prognosis Analysis of Gastrinoma Based on the SEER Database” (Submission ID PONE-D-25-00053). These comments are of great reference value to the revision and improvement of our paper and have important guiding significance to our researches. We have studied comments carefully and have made correction. We hope that the revision is acceptable and look forward to hearing from you soon. Revised portion are marked in color in the paper. The main corrections in the paper and the responds to the reviewer’s comments are as flowing:

Journal Requirements:

When submitting your revision, we need you to address these additional requirements.

1.Please ensure that your manuscript meets PLOS ONE's style requirements, including those for file naming. The PLOS ONE style templates can be found at

https://journals.plos.org/plosone/s/file?id=wjVg/PLOSOne_formatting_sample_main_body.pdf and

https://journals.plos.org/plosone/s/file?id=ba62/PLOSOne_formatting_sample_title_authors_affiliations.pdf

Response: We have carefully reviewed and ensured that our manuscript and all supporting files now fully comply with PLOS ONE's style and formatting requirements.

2. Thank you for stating the following financial disclosure:

“This work was supported by the Science and Technology Development Program of Suzhou (SYWD2024077), the Development Fund of Xuzhou Medical University Affiliated Hospital (XYFZ202404 and XYFM202426), the Program for the Talents in Science and Education of Wujiang District, Suzhou, China (Grant No. WWK202117) and the Scientific Research Project of Suzhou Ninth People’s Hospital (YK202117 and YK202438). There was no additional external funding received for this study.”

Please state what role the funders took in the study. If the funders had no role, please state: "The funders had no role in study design, data collection and analysis, decision to publish, or preparation of the manuscript."

If this statement is not correct you must amend it as needed.

Please include this amended Role of Funder statement in your cover letter; we will change the online submission form on your behalf.

Response: This work was supported by the Science and Technology Development Program of Suzhou (SYWD2024077), the Development Fund of Xuzhou Medical University Affiliated Hospital (XYFZ202404 and XYFM202426), the Program for the Talents in Science and Education of Wujiang District, Suzhou, China (Grant No. WWK202117) and the Scientific Research Project of Suzhou Ninth People’s Hospital (YK202117 and YK202438). There was no additional external funding received for this study. The funders had no role in study design, data collection and analysis, decision to publish, or preparation of the manuscript. We include this amended Role of Funder statement in our cover letter.

3. Please note that funding information should not appear in any section or other areas of your manuscript. We will only publish funding information present in the Funding Statement section of the online submission form. Please remove any funding-related text from the manuscript.

Response: We have removed all funding-related information from the manuscript as requested.

4. Thank you for uploading your study's underlying data set. Unfortunately, the repository you have noted in your Data Availability statement does not qualify as an acceptable data repository according to PLOS's standards.

At this time, please upload the minimal data set necessary to replicate your study's findings to a stable, public repository (such as figshare or Dryad) and provide us with the relevant URLs, DOIs, or accession numbers that may be used to access these data. For a list of recommended repositories and additional information on PLOS standards for data deposition, please see https://journals.plos.org/plosone/s/recommended-repositories.

Response: We have uploaded our study's complete minimal dataset as Supporting Information files alongside the revised manuscript.

We have updated the Data Availability Statement to: Data Availability Statement: All relevant data are within the paper and its Supporting Information files.

5. Please include captions for your Supporting Information files at the end of your manuscript, and update any in-text citations to match accordingly. Please see our Supporting Information guidelines for more information: http://journals.plos.org/plosone/s/supporting-information.

Response: We have now included the captions for all Supporting Information files at the end of the manuscript.

6. Please remove all personal information, ensure that the data shared are in accordance with participant consent, and re-upload a fully anonymized data set.

Note: spreadsheet columns with personal information must be removed and not hidden as all hidden columns will appear in the published file.

Additional guidance on preparing raw data for publication can be found in our Data Policy (https://journals.plos.org/plosone/s/data-availability#loc-human-research-participant-data-and-other-sensitive-data) and in the following article: http://www.bmj.com/content/340/bmj.c181.long.

Response: We have removed all personal information and re-uploaded a fully anonymized dataset.

7. If the reviewer comments include a recommendation to cite specific previously published works, please review and evaluate these publications to determine whether they are relevant and should be cited. There is no requirement to cite these works unless the editor has indicated otherwise.

Response: Thank you for your guidance regarding the reviewer's recommendation to cite specific previously published works. We have carefully reviewed and evaluated the suggested publications, and we confirm that the two additional references requested by Reviewer 2 are highly relevant to our study. As a result, we have incorporated these references into our manuscript to strengthen the context and support our findings. We appreciate the opportunity to enhance the scholarly rigor of our work through this additional citation.

Additional Editor Comments:

Please revise your MS, according to reviewers’suggestions. Some of important publicatons concerned with gastrinoma should be carefully read before writing your MS. In Jensen's review (R. Jensen Ann. Oncol. 1999; 10: Suppl.4, S170-S176), the most common location of gastrinoma was duodenum (about 70%) but in your MS, most of gastrinomas located in pancreas (52.5%) while only 32.5% of gastrinomas located in duodenum, such differences should be discusse.

Response: Thank you for your insightful feedback and for highlighting the discrepancy between our findings and those reported by Jensen et al. We acknowledge the importance of discussing this difference and will revise our manuscript to include a detailed analysis of the potential reasons behind the varying prevalence of gastrinoma locations.

**Revised manuscript:**

Jensen et al. [34] discovered that the duodenum was the most frequent site for gastrinomas, accounting for roughly 70% of cases. In contrast, our study revealed that the pancreas was the predominant location, with 52.5% of gastrinomas found there, while only 32.5% were located in the duodenum. This discrepancy may stem from the fact that Jensen RT's 1999 data were likely influenced by referral biases and the limitations of diagnostic tools available at the time, which led to an overrepresentation of duodenal gastrinoma cases. Our SEER analysis, on the other hand, offers several advantages. It benefits from enhanced pancreatic imaging techniques, a more comprehensive inclusion of non-functional tumors, and population-level sampling. These factors collectively contribute to uncovering a higher true prevalence of pancreatic gastrinomas.

Reviewer's Responses to Questions

Comments to the Author

Reviewer #1: This manuscript presents a population-level analysis of gastrinoma patients using the SEER database from 2000 to 2020. The study aims to assess clinicopathological features and survival outcomes, as well as to compare duodenal and pancreatic gastrinomas. Given the rarity of gastrinomas, this research adds valuable epidemiologic insight and provides potentially practice-relevant data regarding prognosis by tumor location.

**Comment 1：**

The study addresses a rare and clinically important topic using a relatively large dataset for such tumors. By comparing duodenal and pancreatic gastrinomas and identifying independent prognostic factors for overall and cancer-specific survival, the manuscript offers novel findings. This stratified analysis is particularly useful for clinical decision-making.

**Response 1：**

Thank you for your positive feedback and recognition of the clinical significance and novelty of our study. We greatly appreciate your acknowledgment of the stratified analysis and its potential utility in clinical decision-making. Your comments are highly encouraging, and we are glad that our research contributes valuable insights to the field.

**Comment 2：**

Patient selection via ICD-O-3 coding is appropriate.

**Response 2：**

Thank you for your affirmation regarding our patient selection method using ICD-O-3 coding. We appreciate your recognition of its appropriateness.

**Comment 3：**

The use of multiple imputation (MI) for missing data and Cox regression is sound. However, the rationale behind certain cutoff values (e.g., tumor size at 2.0 cm and 5.0 cm) should be clarified.

**Response 3：**

Thank you for your positive assessment of our use of multiple imputation and Cox regression for handling missing data and analyzing survival outcomes. We appreciate your feedback regarding the need to clarify the rationale behind the cutoff values for tumor sizes. The tumor size cutoffs of 2.0 cm and 5.0 cm were chosen based on a combination of clinical relevance and statistical considerations. These specific thresholds align with commonly used clinical benchmarks in the staging and management of neuroendocrine tumors, including gastrinomas. Additionally, these cutoffs allowed for meaningful stratification of patients into distinct prognostic groups, as evidenced by the significant differences in survival outcomes observed in our analysis. We believe these cutoffs provide a balanced and clinically interpretable approach to categorizing tumor sizes in our study.

**Revised manuscript:**

Tumor size was grouped into ≤2.0, 2.1-5.0, and >5.0 cm to reflect clinical stages, the risk of metastasis, and prognostic differences.

**Comment 4：**

While MI is mentioned, the exact method (e.g., number of imputations, convergence diagnostics) should be described in more detail.

**Response 4：**

We appreciate the reviewer’s insightful comment regarding the MI method. In response, we have incorporated more detailed information about the MI process into  the “Statistical analysis” section.

**Revised manuscript:**

Categorical variables were expressed as frequencies and percentages, and Chi-quare tests were employed to evaluate differences among groups. For continuous data that did not follow a normal distribution, the median and interquartile range (IQR) were reported, and comparisons were made using the Mann - Whitney U test. To address missing data in the SEER database, we applied multiple imputation (MI) using the mice package (version 4.1.0) in R. Specifically, we generated 20 imputed datasets by utilizing logistic regression for categorical variables. We assessed convergence both visually through trace plots and statistically using the Gelman-Rubin diagnostic. These assessments confirmed that the imputation models had achieved convergence. The pooled results from these 20 imputed datasets were then used in subsequent analyses to enhance robustness and reduce bias resulting from missing data. For the survival analysis of both OS and CSS, we applied the Kaplan-Meier method. Survival curves were compared using the log-rank test. Additionally, Cox proportional hazards models were used for both univariate and multivariate analyses to calculate hazard ratios (HRs) along with their corresponding 95% confidence intervals (CIs). Variables with a P-value less than 0.05 in the univariate analysis were included in the multivariate Cox regression model. A univariate analysis was carried out to identify potential risk factors associated with liver metastasis. Factors with a P-value below 0.05 from this univariate analysis were subsequently analyzed using a multivariate logistic regression model. All statistical calculations were performed using R software (version 4.1.0). Statistical significance was defined as a P-value less than 0.05.

**Comment 5：**

The use of both univariate and multivariate Cox and logistic regression models is appropriate.

**Response 5：**

Thank you for recognizing the appropriateness of employing both univariate and multivariate Cox and logistic regression models in our study. We appreciate your endorsement of our analytical approach.

**Comment 6：**

Kaplan-Meier curves, log-rank tests, and hazard ratios are well presented.

**Response 6：**

Thank you for acknowledging the clear presentation of Kaplan-Meier curves, log-rank tests, and hazard ratios. We appreciate your positive feedback on our data visualization and analysis.

**Comment 7：**

Several hazard ratios have wide confidence intervals, particularly for cancer-specific survival (e.g., HR = 10.70), likely due to small sample sizes. These should be acknowledged and discussed.

**Response 7:**

We acknowledge the reviewer’s observation regarding the wide confidence intervals observed for several hazard ratios, particularly in relation to cancer-specific survival (e.g., HR = 10.70). We agree that this is likely attributable to the relatively small sample size in our study. We will address this point in the Discussion section of our paper, explicitly acknowledging the limitations imposed by our sample size and its potential impact on the precision of our hazard ratio estimates.

**Revised manuscript:**

Firstly, the sample size of 160 gastrinoma patients is relatively small, which may impact the reliability and precision of our findings, particularly evidenced by the wide CIs observed for some HRs, such as the CSS HR of 10.70 (95% CI 2.55-44.90). This limitation underscores the need for caution in interpreting these results and highlights the importance of larger, multi-center studies to validate our findings. However, it is worth mentioning that gastrinomas are rare tumors globally, and our study includes one of the larger populations of gastrinoma patients at the population level to date.

**Comment 8：**

Language and Writing Quality is moderate — Revision Required. The authors are strongly encouraged to seek professional English language editing to enhance readability and flow.

**Response 8:**

We appreciate the reviewer’s feedback on the language and writing quality of our manuscript. We acknowledge the need for improvement in readability and flow and will seek professional English language editing services to refine the manuscript accordingly. We are committed to ensuring the final version is clear, concise, and accessible to a broad audience. Thank you for your constructive suggestion.

**Comment 9：**

Ethical approval is not required for SEER data analysis. This is correctly stated.

**Response 9:**

Thank you for acknowledging that ethical approval is not required for SEER data analysis and for confirming our correct statement on this matter. We appreciate your attention to detail.

**Comment 10：**

There is no identifiable patient data and the authors have declared no competing interests.

**Response 10:**

Thank you for confirming that no identifiable patient data were used and for acknowledging our declaration of no competing interests. We appreciate your thorough review.

**Comment 11：**

Data availability via the SEER database is clearly stated and in accordance with PLOS ONE requirements.

**Response 11:**

Thank you for recognizing that our data availability statement via the SEER database is clear and meets PLOS ONE’s requirements. We appreciate your attention to compliance and transparency.

**Comment 12：**

Strengths of the Study:

Use of a population-based dataset (SEER) for a rare tumor. Comparison of anatomical subtypes with survival implications. Identification of independent prognostic factors for OS and CSS.

**Response 12:**

Thank you for highlighting the strengths of our study, particularly the use of a population-based SEER dataset for a rare tumor, the meaningful comparison of anatomical subtypes with survival implications, and the identification of independent prognostic factors for OS and CSS. We appreciate your recognition of these key contributions.

**Comment 13：**

Weaknesses and Suggestions for Improvement

Language issues - Manuscript requires grammatical editing and stylistic refinement.

MI explanation - Add detail regarding multiple imputation methodology.

Clinical limitations - Acknowledge the lack of data on recurrence, chemotherapy type, or surgical details in SEER.

Generalizability - Clearly state that SEER-based findings may not generalize beyond the U.S. system.

**Response 13:**

Thank you for your insightful feedback and constructive suggestions. We have addressed each point as follows:

**Language Issues:** We acknowledge the need for grammatical editing and stylistic refinement. The manuscript will undergo a thorough revision by a professional English language editor to ensure clarity, coherence, and adherence to academic writing standards.

**MI Explanation:** We will provide additional details regarding the MI methodology in the Materials and Methods section. Specifically, we will clarify the number of imputations performed, the variables included in the imputation model, and the software used for implementation. This will enhance transparency and reproducibility.

**Clinical Limitations:** We agree that the lack of data on recurrence, chemotherapy type, or surgical details in the SEER database is a limitation. To address this, we will revise the Discussion section to explicitly acknowledge these gaps and their potential impact on the interpretation of our findings. For example: "Third, the SEER database lacks detailed information on postoperative complications, specific chemotherapy regimens, disease recurrence, and the criteria for selecting surgical interventions for individual patients, which may limit the generalizability of our findings regarding treatment outcomes."

**Generalizability:** We will clarify in the Discussion section that the SEER-based findings may not generalize beyond the U.S. healthcare system due to potential variations in clinical practices, diagnostic criteria, and population demographics. For example: "It is important to note that the SEER database primarily reflects the American healthcare system, and our findings may not be directly generalizable to other regions or cultural contexts with differing clinical practices."

Reviewer #2:

**Comment 1：**

1. The purpose of this study was to analyze the clinicopathological features and prognosis of patients with gastrinoma using the SEER database. The chi-square test, Kaplan–Meier method, and Cox proportional hazards model were employed for data analysis, yielding valuable insights. For instance, among 160 patients with gastrinoma, prevalence increased with age, peaking in the 60–69 year group. The pancreas was identified as the most common site of occurrence, and pancreatic gastrinomas were associated with a poorer prognosis compared to those originating in the duodenum. The research methodology was sound and effectively addressed the study’s objectives.

**Response 1:**

Thank you for your positive feedback and recognition of our study’s objectives and methodology. We appreciate your acknowledgment of the valuable insights gained from analyzing the clinicopathological features and prognosis of gastrinoma patients using the SEER database. Your endorsement encourages us to further refine and disseminate our findings.

**Comment 2：**

2. The availability of the original data could further strengthen the support for the conclusions. Additionally, the inclusion of surgery and chemotherapy in the prognostic analysis may warrant further discussion. For example, some patients missed surgical opportunities due to metastasis, while others did not require chemotherapy after curative resection. Given the complexity of these influencing factors, the results are challenging to interpret, a point that was not adequately addressed in the discussion section.

**Response 2:**

We sincerely thank the reviewer for raising these important points regarding data availability and the interpretation of treatment-related factors in our prognostic analysis. We have carefully considered these comments and have revised the manuscript accordingly to provide further clarification and discussion.

As noted in the Data Availability section, the data used in this study are publicly available from the SEER database ([https://seer.cancer.gov/](https://seer.cancer.gov/)). Due to privacy and ethical restrictions, we are unable to share individual-level patient data directly. However, the aggregated data supporting the findings of this study are included within the article. We have now explicitly stated this in the revised manuscript to enhance transparency.

We agree with the reviewer that the roles of surgery and chemotherapy in gastrinoma prognosis are complex and influenced by factors such as tumor stage, metastasis, and individual patient eligibility. To address this, we have expanded the Discussion section to include a more nuanced interpretation of these results. Specifically, we now acknowledge that: Patients with advanced disease or metastasis may have been deemed ineligible for surgery, which could confound the observed association between surgery and improved survival. Similarly, chemotherapy was primarily administered in cases of malignancy or metastasis, which may explain its association with poorer outcomes in multivariate analysis, as it serves as a marker of advanced disease rather than a causative factor for worse prognosis.

We have added the following paragraph to the Discussion section to reflect these considerations: It is important to note that the associations between surgical intervention, chemotherapy, and survival outcomes should be interpreted with caution. Patients who underwent surgery likely had less advanced disease or were deemed suitable candidates for resection, whereas those with metastatic or unresectable tumors may have been excluded from surgical options. Similarly, chemotherapy was predominantly administered in cases with malignant behavior or metastasis, which may explain its association with poorer survival in multivariate analysis. These factors highlight the potential for confounding by indication, and future prospective studies are needed to better elucidate the causal effects of these treatments on gastrinoma prognosis.

We believe these revisions provide a more balanced and critical discussion of the treatment-related findings and acknowledge the limitations inherent in retrospective database analyses. Thank you again for these insightful comments, which have significantly improved the clarity and depth of our manuscript.

**Comment 3：**

3. Gastrinomas are rare tumors; however, for a case series spanning 20 years, the number of cases remains relatively small. Larger prospective studies on gastrinoma have already been reported earlier. Moreover, among the 160 gastrinoma cases analyzed in this study, how many were sporadic and how many were MEN-1-related? MEN-1-associated gastrinoma patients typically present at a younger age, and some studies suggest this may correlate with prognosis. If the gastrinoma cases in this study include both sporadic and MEN-1-related types, it is recommended to discuss them separately. As this study and the prior work by Robert T. Jensen et al. both pertain to US patient populations, it would be valuable to explicitly compare the clinical manifestations and prognosis with those findings, and differences should be analized and discussed.

References:

(1) Yu F, et al. J Clin Oncol. 1999;17(2):615-30.

(2) Gibril F, et al. Medicine (Baltimore). 2004;83(1):43-83.

**Response 3:**

Thank you for your insightful and professional comments. We greatly appreciate your thorough review and valuable suggestions, which have helped us to further improve our manuscript.

You rightly pointed out that gastrinomas are rare tumors, and the sample size of 160 cases over a 20-year period remains relatively limited. We acknowledge that larger prospective studies on gastrinoma have been conducted previously. However, as a population-based study utilizing the SEER database, our research provides a broad overview of the clinicopathological characteristics and prognosis of gastrinoma within a U.S. cohort, which we believe still offers meaningful insights despite the sample size constraints.

Regarding the important distinction between sporadic and MEN-1-related gastrinomas, we agree that this is a critical factor influencing clinical presentation and prognosis, particularly given that MEN-1-associated cases often present at a younger age and may have different outcomes. Unfortunately, the SEER database does not include specific information on MEN-1 status or allow for differentiation between sporadic and hereditary cases. This is indeed a limitation of our study. We agree that future studies should strive to incorporate genetic and syndromic information to enable more nuanced analyses.

In response to your suggestion, we have expanded the Discussion to include a comparative analysis with previous U.S.-based studies, particularly those by Robert T. Jensen and colleagues, regarding clinical manifestations and prognosis. We have highlighted similarities and differences in age distribution, tumor location, survival outcomes, and other key clinicopathological features, and have provided potential explanations for these discrepancies.

Thank you once again for your constructive feedback, which has significantly strengthened our manuscript.

**Revised manuscript:**

Our population-based study reveals several key findings that both corroborate and contrast with prior landmark studies by Yu et al. [35] and Gibril et al. [36]. Regarding age distribution, our SEER data showed peak incidence at 60-69 years, which aligns with Yu et al.'s cohort (median age 50 years at diagnosis) but suggests a later presentation compared to Gibril et al.'s MEN1-associated cases (median age 38 years). This discrepancy likely reflects our inclusion of sporadic gastrinomas (77.5% well-differentiated), whereas Gibril et al.'s cohort predominantly studied MEN1-related tumors known for earlier onset. Tumor location patterns in our study (pancreas 52.5%, duodenum 32.5%) differ notably from both references. Yu et al. reported duodenal predominance (70%), while Gibril et al. found pancreatic lesions in 55% of sporadic cases. Our higher pancreatic proportion may reflect improved imaging detection of small duodenal tumors in modern eras. Crucially, we confirmed duodenal tumors' superior prognosis (5-year CSS 86% vs pancreatic 75.2%), mirroring Yu et al.'s observation of better survival with duodenal lesions, though their cohort showed lower 10-year survival (51% overall) compared to our 62.9% OS - potentially due to advances in metastatic disease management. Notably, our multivariate analysis identified tumor size and N stage as independent prognostic factors, consistent with Yu et al.'s emphasis on metastatic burden. However, while Yu et al. reported liver metastases as the dominant cause of death (50%), our SEER data showed better metastatic control (5-year CSS 86%), possibly attributable to increased surgical resection rates (49% enucleation/36% pancreatic resection in comparable studies) and somatostatin analogs introduced post-2000.

36. Yu F, Venzon DJ, Serrano J, et al. Prospective study of the clinical course, prognostic factors, causes of death, and survival in patients with long-standing Zollinger-Ellison syndrome. J Clin Oncol. 1999 Feb;17(2):615-30. doi: 10.1200/JCO.1999.17.2.615. PMID: 10080607.
37. Gibril F, Schumann M, Pace A, et al. Multiple endocrine neoplasia type 1 and Zollinger-Ellison syndrome: a prospective study of 107 cases and comparison with 1009 cases from the literature. Medicine (Baltimore). 2004 Jan;83(1):43-83. doi: 10.1097/01.md.0000112297.72510.32. Erratum in: Medicine (Baltimore). 2004 May;83(3):175. PMID: 14747767.

**Comment 4：**

4. Several language issues were noted that affect clarity and precision. For instance, in the “Results — Patient Characteristics” section, the sentence “A study involving 160 patients revealed that the incident rate was followed with age” should replace “incident rate” with “prevalence.” Attention should also be paid to consistency in tense, punctuation, numerical formatting, and terminology throughout the manuscript.

**Response 4:**

Thank you for this valuable feedback. We sincerely appreciate you taking the time to identify these language issues, which we agree are essential for the clarity and precision of the manuscript. We will carefully revise the entire manuscript to address the points you have raised. We are committed to improving the overall readability and linguistic quality of the paper and believe these revisions will significantly enhance the manuscript.

**Comment 5：**

5. Furthermore, improving the clarity of figures is recommended. For example, in Figure 2A and Figure 2B, which depict Overall Survival (OS) and Cancer-Specific Survival (CSS) of gastrinoma patients, respectively, the titles should explicitly indicate “OS” and “CSS” to facilitate reader comprehension.

**Response 5:**

Thank you for this helpful suggestion. We agree that improving the clarity of the figures will enhance the reader's understanding. We will revise Figure 2A and Figure 2B to explicitly include "Overall Survival (OS)" and "Cancer-Specific Survival (CSS)" in their respective titles, as recommended. We appreciate your valuable feedback for improving the presentation of our manuscript.

Yours sincerely,
