# Supplementary material for: Clinicopathological characteristics and prognosis analysis of gastrinoma based on the SEER database
Source: PLoS One. 2025 Dec 17;20(12):e0337940. doi: 10.1371/journal.pone.0337940 (PMC12711040; doi:10.1371/journal.pone.0337940)
Supplement: Attachment — Submitted filename: Point by Point Response R2.docx [file pone.0337940.s004.docx]

Dear editor and reviewers

Submission ID PONE-D-25-00053

Title: Clinicopathological Characteristics and Prognosis Analysis of Gastrinoma Based on the SEER Database

By: zhenguo qiao et al

Thank you very much for your letter and for the editors’ and reviewers’ comments concerning our manuscript entitled “Clinicopathological Characteristics and Prognosis Analysis of Gastrinoma Based on the SEER Database” (Submission ID PONE-D-25-00053). These comments are of great reference value to the revision and improvement of our paper and have important guiding significance to our researches. We have studied comments carefully and have made correction. We hope that the revision is acceptable and look forward to hearing from you soon. Revised portion are marked in color in the paper. The main corrections in the paper and the responds to the reviewer’s comments are as flowing:

Reviewer #2: I thank the authors for their comprehensive response to the previous comments. There are just a few minor points that still require attention.

**Comment 1：**

1. Regarding the stratification of tumor size, the ENET guidelines utilize categories of ≤2 cm, 2-4 cm, and >4 cm. Please consider whether adopting this established criteria would be appropriate for your study.

**Response 1：**

We sincerely thank the reviewer for this valuable and insightful suggestion. We agree that aligning our analysis with the established ENETS guideline criteria for tumor size stratification (≤2 cm, 2-4 cm, and >4 cm) will enhance the clinical relevance and interpretability of our findings, and we appreciate the reviewer pointing this out. In accordance with this comment, we have now re-stratified the tumor size variable in our dataset using the recommended categories: ≤2 cm, 2.1-4 cm, and >4 cm. Subsequently, we have repeated all relevant statistical analyses, including: 1) The comparison of clinicopathological characteristics (now reflected in the updated Table 1 and Table 3). 2) The univariate and multivariate Cox regression analyses for Overall Survival (OS) and Cancer-Specific Survival (CSS) (now presented in the revised Table 4 and Table 5). 3) The univariate and multivariate analysis of risk factors for liver metastasis (now shown in the updated Table 6). Thank you once again for this constructive comment, which has undoubtedly improved the quality of our manuscript.

**Comment 2：**

2. I appreciate the authors' explanation for treatment-related factors in the prognostic analysis. From the presented results, both surgery and chemotherapy appear to be protective factors. However, the discussion states, "Similarly, chemotherapy was predominantly administered in cases with malignant behavior or metastasis, which may explain its association with poorer survival in multivariate analysis." This seems to indicate chemotherapy is associated with poorer survival. Please verify this apparent inconsistency.

**Response 2：**

We sincerely thank the reviewer for this exceptionally careful and astute observation regarding the interpretation of the chemotherapy results. The reviewer is absolutely correct to point out the apparent inconsistency between our multivariate analysis results, which show chemotherapy as a protective factor (as indicated by HR > 1 for the "No/Unknown" group in Tables 4 and 5), and our discussion statement, which suggested an association with poorer survival. We apologize for this confusion and the oversight in our manuscript wording. The results presented in Tables 4 and 5 are accurate. In our Cox regression model, the "No/Unknown" chemotherapy group was used as the reference. A Hazard Ratio (HR) greater than 1 for this reference group indeed indicates that receiving chemotherapy ("Yes" group) is associated with a lower risk of death, meaning it appears as a protective factor in the statistical model. However, this statistical finding is almost certainly subject to significant confounding by indication. As we attempted to discuss, patients who received chemotherapy were likely those with more aggressive or advanced disease at baseline. The protective HR likely reflects that, despite having worse prognostic factors, these patients' survival was not as poor as it would have been without chemotherapy, hence the model attributes a "protective" effect to the treatment. Our statement in the discussion that "chemotherapy was predominantly administered in cases with malignant behavior or metastasis, which may explain its association with poorer survival" was incorrectly phrased and created the inconsistency. We intended to explain the underlying clinical context that biases the raw statistical association, but we erroneously used "poorer survival" instead of describing the confounding nature of the indication.

Actions Taken:
We have thoroughly revised the relevant section in the Discussion to remove this contradictory statement and to provide a clearer and more accurate interpretation. The revised text now reads: "It is important to note that the associations between surgical intervention, chemotherapy, and survival outcomes should be interpreted with caution. The protective effects observed in our multivariate analysis are likely influenced by confounding by indication. Patients who underwent surgery or received chemotherapy were a selected group; surgical candidates typically had less advanced or resectable disease, while chemotherapy was predominantly administered in cases with more aggressive tumor behavior or metastasis. Therefore, the observed hazard ratios may not represent the true causal effects of these treatments, and prospective studies are needed for definitive evaluation." Thank you once again for highlighting this critical issue, which has allowed us to improve the clarity and accuracy of our manuscript.

**Comment 3：**

3. This study identifies the pancreas as a common site for gastrinomas. Therefore, should the statement, "Our higher pancreatic proportion may reflect improved imaging detection of small duodenal tumors in modern eras," be revised to suggest that it reflects improved detection of pancreatic tumors instead?

**Response 3：**

We sincerely thank the reviewer for this excellent and logical suggestion. The reviewer is absolutely correct. Our original statement, which suggested that a higher proportion of pancreatic gastrinomas might be due to improved detection of duodenal tumors, was indeed counterintuitive and incorrectly phrased. We fully agree with the reviewer's reasoning. The more plausible explanation, which aligns with our central finding of the pancreas being the most common site, is that advancements in cross-sectional imaging (such as CT and MRI) have particularly enhanced the detection of pancreatic tumors in the modern era.

We have revised the sentence in the Discussion section accordingly. The original sentence: "Our higher pancreatic proportion may reflect improved imaging detection of small duodenal tumors in modern eras." Has been changed to: "Our higher pancreatic proportion may reflect improved imaging detection of pancreatic tumors in the modern era." Thank you again for this valuable correction, which significantly improves the logical consistency of our discussion.

Reviewer #3: Gastrinomas are rare tumors classified as a type of neuroendocrine tumor. This study conducted clinicopathological and prognostic analyses based on 20-year case data from the SEER database, featuring a relatively large sample size, comprehensive data collection, and appropriate analytical methods. Ultimately, multivariate analysis revealed that pancreatic-origin gastrinomas have a poor prognosis, and tumor grade, N stage, surgery, chemotherapy, and tumor size are independent risk factors affecting the cancer-specific survival (CSS) of gastrinoma patients, which holds certain reference value for clinical practice.

Due to its reliance on retrospective data from the SEER database, the study lacks data on clinical manifestations, gastrin levels, pathological Ki-67 index, and SSTR2 immunohistochemical results, which may introduce some bias into the findings. However, this is a common limitation of such retrospective studies. If data on this type of tumor from local institutions is combined with SEER database data for further analysis, the research results will be more reference-worthy.

**Response ：**

We are sincerely grateful to the reviewer for their positive assessment of our work and for providing constructive and insightful suggestions to further strengthen our manuscript. We completely agree with the reviewer that the lack of detailed clinical and pathological data, such as clinical symptoms, serum gastrin levels, Ki-67 index, and SSTR2 expression status, is an important limitation of our study based on the SEER database. As the reviewer rightly pointed out, this is an inherent constraint of large-scale registry data, but incorporating these variables would undoubtedly provide a more comprehensive understanding of gastrinoma. In response to this valuable comment, we have added a statement to the Limitations section of our Discussion to explicitly acknowledge this point. The added text reads: "Furthermore, as a registry-based study, the SEER database lacks detailed information on clinical presentations, serum gastrin levels, and key pathological markers such as the Ki-67 proliferation index and somatostatin receptor (SSTR) expression status. The inclusion of these clinically significant parameters could provide deeper insights into tumor behavior and prognostication." Once again, we thank the reviewer for this excellent suggestion. We believe that addressing this limitation has enhanced the transparency and scholarly rigor of our manuscript. We fully endorse the reviewer's view that future studies combining institutional data with larger databases will be highly valuable.

Yours sincerely,
